# Understanding International Differences in Academic Author Order in General Medicine Publications

**DOI:** 10.14789/jmj.JMJ23-0012-P

**Published:** 2023-07-24

**Authors:** MIWA SEKINE, YASUHIKO KIYAMA, RIEKO UEDA, DAVID AUNE, SHOJI SANADA, YUJI NISHIZAKI

**Affiliations:** 1 Division of Medical Education, Juntendo University Faculty of Medicine, Tokyo, Japan; 1 Division of Medical Education, Juntendo University Faculty of Medicine, Tokyo, Japan; 2 Hongo-Ochanomizu Campus Academic Media Center, Juntendo University, Tokyo, Japan; 2 Hongo-Ochanomizu Campus Academic Media Center, Juntendo University, Tokyo, Japan; 3 Clinical Research and Trial Center, Juntendo University Hospital, Tokyo, Japan; 3 Clinical Research and Trial Center, Juntendo University Hospital, Tokyo, Japan; 4 Clinical and Translational Research Center, Kobe University Hospital, Tokyo, Japan; 4 Clinical and Translational Research Center, Kobe University Hospital, Tokyo, Japan; 5 Clinical Translational Science, Juntendo University Graduate School of Medicine, Tokyo, Japan; 5 Clinical Translational Science, Juntendo University Graduate School of Medicine, Tokyo, Japan

**Keywords:** authorship, research, academia, publishing

## Abstract

**Objectives:**

With increasing multinational research in general medicine, the lack of a standardized policy regarding the order of author bylines can create conflict and misunderstanding due to different practices worldwide.

**Methods:**

We examined publicly available data from websites such as Journal Citation Reports and Web of Science, focusing on original articles published in the “Medicine, General, & Internal” category in 2020. Of 169 journals in the “Medicine, General, & Internal” category, we selected the ten countries with the highest number of publications and then examined the position of the corresponding author in the author byline as an indicator of the author in charge since corresponding authors are considered to have contributed the most.

**Results:**

The top ten countries with the highest publications are the USA, China, Germany, England, Japan, France, Italy, Canada, India, and Australia. The results demonstrated that the percentage of the second author being the corresponding author was the highest in Japan compared to other countries. This percentage was 25 times higher in Japan than in the USA.

**Conclusions:**

Understanding international differences regarding author order would facilitate smoother collaboration.

## Introduction

Recently, the importance of clinical research has been increasing. However, general practitioners or primary care physicians' contributions to clinical research have been somewhat limited compared to other specialists^1)^, particularly in Japan^2)^. Historically, the role of primary care physicians has been defined by WHO as first-contact, accessible, continuous, comprehensive, and coordinated person-focused care, and is a key part of primary health care in achieving the goals stated in the “Health For All” in the Alma Ata Declaration^3)^. Owing to the establishment of general medicine as a medical board subspecialty in 2018^4)^, the research contributions of primary care physicians are increasing, especially from younger primary care physicians.

Subsequently, it is imperative to promote high-quality and credible evidence-based research designs and international collaborations to conduct high-quality clinical research. Training and educating principal investigators play a major role in research institutions^5)^, but the intricacy of unspoken rules regarding the author order (e.g., first author, last author) are not covered in such training. In many countries, the order of authors in academic publications is as important as the impact factor of the journal in which they publish^6)^. With increasing multinational research, the lack of a standardized policy regarding author order can create conflict and misunderstanding due to differing practices worldwide, hindering smooth collaboration^7)^. Here, we studied author order in publications in different countries.

## Methods

In this study, we used publicly available data from websites such as Journal Citation Reports and Web of Science, focusing on original articles published in 2020.

### Discipline selection

Of the 178 fields registered in the Journal Citation Reports database, we selected “Medicine, General, & Internal” as our target discipline with 169 journals and examined the number of articles. We then selected the ten countries with the highest number of publications in the Web of Science: Science Citation Index Expanded database published from 2011 to 2020. We compared the number of publications in 2020 by country.

### Order of author criteria

The corresponding authors are considered to have contributed the most since they are the primary correspondents with editorial offices and are responsible for decisions regarding manuscripts^8)^. Therefore, we chose the corresponding author as an indicator of the author in charge. To determine the perceived credit of author order, we categorized the position in which corresponding authors are likely to be listed in articles such as 1) first author, 2) second author, 3) third author, 4) penultimate author, 5) last author, and 6) other position. We also set the following criteria: (1) to differentiate the first, second, penultimate, third, and last authors, we selected articles with more than five authors without group investigators; (2) we also selected articles with one corresponding author with one affiliated institution; and (3) we selected articles with the corresponding author affiliation and journal from the same country to evaluate the characteristics of countries with minimal influence from other countries. We then examined the position of the corresponding author in the author byline and compared countries.

## Results

The top ten countries with the highest publications are the USA, China, Germany, England, Japan, France, Italy, Canada, India, and Australia (Table 1). Of the top ten countries, the USA, China, England, Japan, France, Italy, Canada, and India met the study's criteria. Seven journals were published in English, and three were published in the native language of the journal's country. The comparison of the corresponding author position by country is illustrated in Table 2. The percentage of second authors being corresponding authors by country was as follows: Canada = 1.8%, China = 3.8%, England = 4.4%, France = 6.8%, India = 18.6%, Italy = 18.2%, Japan = 30.2%, and the USA = 1.2% (Table 2).

**Table 1:** Number of articles published in 2020

Country	Journal name	Articles published (2020)
Australia	International Journal of Medical Sciences	307
Canada	Canadian Medical Association Journal	86
China	Chinese Medical Journal	193
England	BMJ Open	2,678
France	Revue De Médecine Interne	60
Germany	European Journal of Clinical Investigation	168
India	Indian Journal of Medical Research	96
Italy	Acta Medica Mediterranea	590
Japan	Internal Medicine	454
USA	JAMA Network Open	1,026

Note. The right column shows the number of articles published by journals in the middle column in 2020.

**Table 2:** Summary of the corresponding author position by country

Country	Canada	China	England	France	India	Italy	Japan	USA
Author position	N (%)	N (%)	N (%)	N (%)	N (%)	N (%)	N (%)	N (%)
First	31 (56.4)	7 (4.5)	240 (70.6)	25 (56.8)	14 (23.7)	10 (30.3)	209 (51)	415 (71.4)
Second	1 (1.8)	6 (3.8)	15 (4.4)	3 (6.8)	11 (18.6)	6 (18.2)	124 (30.2)	7 (1.2)
Third	2 (3.6)	4 (2.5)	0 (0)	0 (0)	2 (3.4)	2 (6.1)	12 (2.9)	0 (0)
Penultimate	0 (0)	7 (4.5)	6 (1.8)	1 (2.3)	2 (3.4)	1 (3)	6 (1.5)	6 (1)
Last	21 (38.2)	129 (82.2)	76 (22.4)	15 (34.1)	27 (45.8)	11 (33.3)	56 (13.7)	152 (26.2)
Other	0 (0)	4 (2.5)	3 (0.9)	0 (0)	3 (5.1)	3 (9.1)	3 (0.7)	1 (0.2)
Total	55	157	340	44	59	33	410	581

Note. N represents the number of articles published by each journal in 2020, with articles that did not meet the selection criteria excluded.

## Discussion

In this study of author order in general medicine journals, the results revealed different patterns of author order compared to Japan. The result demonstrated that the percentage of the second author being the corresponding author was the highest in Japan compared to other countries. This percentage was 25 times higher in Japan than in the USA. The second author holding the corresponding author position in Japan was greater than in other countries by the following factors: Canada = 16.6, China = 7.9, England = 6.9, France = 4.4, India = 1.6, Italy = 1.7, and the USA = 25.1. This finding aligns with the previous report that while the number of corresponding authors being first and last authors is higher in France, first and second authors being the corresponding authors is more common in Japan^9)^.

In Japan, laboratory chairpersons supervise Ph.D. students, but their priority is often securing research funds and laboratory management rather than guiding students in daily research. Instead, assistants or associate professors are likely to be the advisors for students. This suggests that the last author position may be reserved for chairpersons who provide research funds.

Although further research is needed, several possibilities can be speculated. First, the corresponding author in charge of directly guiding Ph.D. students' daily work is listed as the last author outside Japan. Second, as suggested above, supervising faculties take a second author position to give the chairperson the last author position. However, several publications as the corresponding author are essential for junior researchers' promotion opportunities, especially in becoming tenured professors in Japan. We speculate that chairpersons might designate junior researchers supervising Ph.D. students' daily research as the second author and corresponding authors for their future carrier path.

This study has several limitations. First, the data used for this study were from 2020, collected during the COVID-19 pandemic. Therefore, it might not be generalizable. Second, our study focused on general internal medicine journals, which may not be generalizable as it may not be a representative sample worldwide, including other clinical fields. Third, we did not differentiate between open-access journals and others, which could skew the results so that authors with funding were more likely to be listed as corresponding authors due to the Article Processing Charge.

In conclusion, academic author order in other disciplines and international journals should be examined further to clarify global norms and explore author order standards. In any case, multinational scientific collaborations are key to scientific breakthroughs and should be encouraged in any discipline or field, especially in medicine. Understanding international norms regarding the order of author bylines would facilitate smoother collaboration, especially in multinational research projects, and clarify the recognition of researchers' contributions.

## Funding

This research received no specific grant from any funding agency in the public, commercial, or not-for-profit sectors.

## Author contributions

YN and SS conceived of the presented idea. YN developed the theory and YK and RU performed the computations. MS verified the analytical methods. MS, DA and YN analyzed and interpreted the data. MS, DA and YN were major contributor in writing the manuscript. YN and SS supervised. All authors discussed the results and contributed to the final manuscript. All authors read and approved the final manuscript.

## Conflicts of interest statement

The authors declare that there are no conflicts of interest.
